# Supplementary material for: Analysis of PPARγ Signaling Activity in Psoriasis
Source: Int J Mol Sci. 2021 Aug 10;22(16):8603. doi: 10.3390/ijms22168603 (PMC8395241; doi:10.3390/ijms22168603)
Supplement: Supplementary file 1 [file ijms-22-08603-s001.zip › Supplemental materials_Analysis of PPARg signaling activity in psoriasis/Pathway models/Models images and html files/Model of PPARG related pathways in psoriasis (short version)/3845.html]

EGFR


# Protein EGFR

|  |  |
| --- | --- |
| URN | urn:agi-llid:1956 |
| Total Entities | 0 |
| Connectivity | 20033 |
| Name | EGFR |
| Description | epidermal growth factor receptor |
| Notes | The protein encoded by this gene is a transmembrane glycoprotein that is a member of the protein kinase superfamily. This protein is a receptor for members of the epidermal growth factor family. EGFR is a cell surface protein that binds to epidermal growth factor. Binding of the protein to a ligand induces receptor dimerization and tyrosine autophosphorylation and leads to cell proliferation. Mutations in this gene are associated with lung cancer. [provided by RefSeq, Jun 2016] |
| Primary Cell Localization | Plasma membrane |
| Class | Receptor |

---

|  |  |
| --- | --- |
| Pathway | Proteins Involved in Stem Cell Exhaustion in Aging |
|  | Skin Aging |
|  | Prolactin Production |
|  | Vascular Smooth Muscle Cell/Pericyte Migration and Proliferation |
|  | NTSR1 Expression Targets |
|  | DRD2 Expression Targets |
|  | DRD2 -> TRPC1 Transcription |
|  | CHRM1 -> IL2 Production |
|  | Luteinizing Hormone in Oocyte Meiotic Resumption |
|  | G0/G1 Phase Transition |
|  | Gap Junction Assembly |
|  | Axonal Transport |
|  | Transcytosis |
|  | Proteins Involved in Atherosclerosis |
|  | Scavenger Receptor OLR1 in Inflammation Related Endothelial Dysfunction |
|  | Proteins Involved in Dilated Cardiomyopathy |
|  | Proteins Involved in Hypertrophic Cardiomyopathy |
|  | Proteins Involved in Arterial Hypertension |
|  | Arterial Hypertension |
|  | Endothelial Cell Dysfunction in Arterial Hypertension |
|  | Proteins Involved in Pulmonary Hypertension |
|  | Smooth Muscle Cell Dysfunction in Pulmonary Hypertension |
|  | Myocardial Remodeling |
|  | Proteins Involved in Myocarditis |
|  | Proteins with Altered Expression in Melanoma |
|  | Melanoma |
|  | Proteins Involved in Melanoma |
|  | Proteins Involved in Psoriasis |
|  | Apoptotic Keratinocytes Clearance Recession in Systemic Lupus Erythematosus |
|  | Proteins Involved in Diabetic Nephropathy |
|  | Hepatic Stellate Cells in non-Alcoholic Fatty Liver Disease |
|  | beta-Cell Mass Regulation |
|  | Prolactinoma Overiew |
|  | Proteins Involved in Colorectal Neoplasms |
|  | Cetuximab Resistance in Colorectal Cancer |
|  | Metastatic Colorectal Cancer |
|  | EGFR Nuclear Signaling in Colorectal Cancer |
|  | OipA Signaling in Helicobacter Pylori Infection |
|  | Apoptosis through VacA in Helicobacter Pylori Infection |
|  | CagA Phosphorylation Dependent Signaling |
|  | Multiple Myeloma |
|  | Proteins Involved in Hepatocellular Carcinoma |
|  | Hepatocellular Carcinoma |
|  | Growth Factor Signaling in Hepatocellular Carcinoma |
|  | Proteins Involved in Pancreatic Cancer |
|  | Pancreatic Ductal Carcinoma |
|  | Growth Factor Signaling in Pancreatic Neoplasms |
|  | Pancreatic Neuroendocrine Tumors |
|  | Goblet-Cell Exocytosis in Asthma |
|  | Goblet-Cell Related Mucus Secretion in Asthma |
|  | Mucin Production in Goblet Airway Epithelial Cells |
|  | Axonal Transport Impairment in Hungtington Disease |
|  | Glioblastoma, Primary |
|  | Glioblastoma, Classical Subtype |
|  | Proteins Involved in Glioblastoma |
|  | Glioblastoma, Secondary |
|  | Astrocytoma |
|  | Proteins Involved in Oligodendroglioma |
|  | Proteins Involved in Astrocytoma |
|  | Glioma Invasion Signaling |
|  | Proteins Involved in Glioma |
|  | Growth Factor Signaling in Neuroblastoma |
|  | Neuroblastoma |
|  | Proteins Involved in Neuroblastoma |
|  | Proteins Involved in Breast Cancer Related to NOTCH Signaling Pathway |
|  | Proteins Involved in Breast Cancer Related to ERBB2/VEGFR/Akt Signaling Pathway |
|  | ERBB/VEGFR/Akt Signaling in Breast Cancer |
|  | ESR1/ERBB Positive Luminal Breast Cancer |
|  | Proteins with Altered Expression in Breast Cancer |
|  | Breast Cancer |
|  | NOTCH1 Signaling in Breast Cancer |
|  | Endometrial Cancer |
|  | Endometrioid Endometrial Cancer |
|  | Clear Cell Endometrial Cancer and Papillary Serous Endometrial Cancer |
|  | Proteins with Altered Expression in Endometrial Cancer |
|  | Proteins Involved in Endometriosis |
|  | Proteins Involved in High-Grade Serous Ovarian Carcinoma |
|  | Endometrioid Ovarian Carcinoma |
|  | Proteins Involved in Endometrioid Ovarian Carcinoma |
|  | Proteins Involved in Mucinous Ovarian Carcinoma |
|  | EGFR and PTAFR Signaling in Ovarian Cancer |
|  | High-Grade Serous Ovarian Carcinoma |
|  | Low-Grade Serous Ovarian Carcinoma |
|  | Proteins Involved in Low-Grade Serous Ovarian Carcinoma |
|  | Ovarian Cancer |
|  | Mucinous Ovarian Carcinoma |
|  | Proteins with Altered Expression in Ovarian Cancer |
|  | EGF/cyclinD1 Signaling in Acquired Middle Ear Cholesteatoma |
|  | Subepithelial Fibroblasts in Middle Ear Cholesteatoma |
|  | Cholesteatoma |
|  | HPV Infection and Cancer |
|  | HPV E5 Regulates EDNRA and EGFR Signaling Pathway in Keratinocytes |
|  | Androgen Receptor Coregulator ARA55 (TGFB1I1) in Prostate Cancer |
|  | Prostate Cancer |
|  | Proteins Involved in Prostate Cancer |
|  | Proteins Involved in Otitis Media |
|  | Cytokines Trigger Otitis Media |
|  | Proteins Involved in Chronic Obstructive Pulmonary Disease |
|  | Mucin Hyperproduction in Goblet and Mucous Cells |
|  | Proteins Involved in Polycystic Kidney Disease |
|  | Proteins Involved in Glomerulonephritis |
|  | Sertoli Cells Dysfunction Causes Male Infertility |
|  | AREG -> NFATC Expression Target |
|  | AREG -> CREB/CREBBP Expression Targets |
|  | AREG -> NCOR2 Expression Targets |
|  | AREG -> SMAD1 Expression Target |
|  | AREG -> CTNN Expression Targets |
|  | AREG -> HIF1A Expression Targets |
|  | AREG -> STAT Expression Targets |
|  | AREG -> AP-1 Expression Targets |
|  | AREG -> FOXO3A Expression Target |
|  | Acetylcholine Expression Targets |
|  | Dopamine/Gi Expression Targets |
|  | Aryl Hydrocarbon Receptor Genomic and non-Genomic Signaling |
|  | BMP4/BMPR2 Expression Targets |
|  | BTC -> NFATC Expression Targets |
|  | BTC -> AP-1/ATF/CREB Expression Targets |
|  | BTC -> CTNN Expression Targets |
|  | BTC -> STAT Expression Targets |
|  | GPCR Family -> Expression Targets in Adipose Tissue |
|  | Receptor Tyrosine Kinase Family -> Expression Targets in Bone |
|  | Elevated Receptors -> Expression Targets in Blood vessel |
|  | Elevated Receptors -> Expression Targets in Myocardium |
|  | Elevated Receptors -> Expression Targets in Aorta |
|  | Elevated Receptors -> Expression Targets in Blood Vessel |
|  | CXCL12 Expression Targets |
|  | CSF2 -> NF-kB Expression Targets |
|  | Tamoxifen Induced Endometrial Cancer |
|  | EGF -> CTNN Expression Targets |
|  | EGF -> HIF1A Expression Targets |
|  | EGF -> AP-1/ATF Expression Targets |
|  | EGF -> CREB/CREBBP/ELK/SRF/MYC Expression Targets |
|  | EGF -> MEF/MYOD/NFATC Expression Targets |
|  | EGF -> FOXO3A Expression Targets |
|  | EGF -> TP53 Expression Targets |
|  | EGF -> NCOR2 Expression Target |
|  | EGF -> STAT Expression Targets |
|  | EGFR -> NCOR2 Signaling |
|  | EGFR/ERBB3 -> MEF/MYOD/NFATC/MYOG Signaling |
|  | EGFR -> CTNND Signaling |
|  | EGFR/ERBB2 -> TP53 Signaling |
|  | EGFR/ERBB2 -> CTNNB Signaling |
|  | EGFR -> SMAD1 Signaling |
|  | EGFR/ERBB2 -> HIF1A Signaling |
|  | EGFR -> ZNF259 Signaling |
|  | EGFR -> AP-1/ATF2 Signaling |
|  | EGFR -> AP-1/CREB/ELK/SRF/MYC Signaling |
|  | EGFR/ERBB -> STAT Signaling |
|  | EREG -> CREB Expression Target |
|  | EREG -> FOXO3A Expression Target |
|  | EREG -> CTNNB/CTNND Expression Target |
|  | EREG -> STAT Expression Targets |
|  | EREG -> HIF1A Expression Target |
|  | EREG -> AP-1/ATF Expression Targets |
|  | Elevated Receptors -> Expression Targets in Endocrine Gands |
|  | F2 -> AP-1/CREB/ELK/SRF/SP1 Expression Targets |
|  | F2 -> STAT1/NF-kB Expression Targets |
|  | PLG -> AP-1/CREB/ELK/SRF/SP1 Expression Targets |
|  | PLG -> STAT1/NF-kB Expression Targets |
|  | FGF2 -> STAT Expression Targets |
|  | FGF7 -> AP-1/CREB/CREBBP/MYC Expression Targets |
|  | FGF2 -> AP-1/CREB/CREBBP/ELK/SRF/MYC Expression Targets |
|  | GCG Expression Targets |
|  | FSHR Expression Targets |
|  | Glutamate/Gq Expression Targets |
|  | VIP Expression Targets |
|  | Elevated Receptors -> Expression Targets in Mouth |
|  | EGFR -> Expression Targets in Colon |
|  | HBEGF -> STAT Expression Targets |
|  | HBEGF -> FOXO3A Expression Target |
|  | HBEGF -> AP-1/ATF Expression Targets |
|  | HBEGF -> TP53 Expression Targets |
|  | HBEGF -> EP300/ETS/ETV/SP1 Expression Targets |
|  | HBEGF -> HIF1A Expression Targets |
|  | HBEGF -> MEF/MYOD Expression Target |
|  | HBEGF -> CREB/MYC Expression Targets |
|  | HGF -> STAT Expression Targets |
|  | HGF -> AP-1/CREB/ELK/SRF/MYC Expression Targets |
|  | EGFR Transactivation in Cancer and Non-Cancer Cells |
|  | TAM Receptor Signaling Activation in Cancer |
|  | Receptors and Adaptor Proteins Activated in Cancer |
|  | Proteins with Altered Expression in Cancer-Associated Sustaining of Proliferative Signaling |
|  | Genes with Mutations in Cancer-Associated Sustaining of Proliferative Signaling |
|  | G0/G1 Cell Cycle Phase Transition Activation in Cancer |
|  | STAT3 and NFkB Activate Inflammation-Induced Tumorigenesis |
|  | VEGFA Dependent Angiogenesis in Cancer |
|  | Integrins in Cancer Cell Motility, Invasion and Survival |
|  | Proteins with Altered Expression in Cancer Metastases |
|  | Epithelial to Mesenchymal Transition in Cancer: Overview |
|  | BSG (CD147) in Cancer Cells Motility, Invasion and Survival |
|  | Hyaluronic Acid, CD44 and HMMR in Cancer Cell Invasion and Survival |
|  | Invadopodia Formation in Cancer Cells |
|  | Lipogenesis/Lipolysis Activation in Cancer Cells and Cancer-Associated Adipocytes |
|  | Genes with Mutations in Cancer Metabolic Reprogramming |
|  | Proteins with Altered Expression in Cancer Metabolic Reprogramming |
|  | IGF1 -> MEF/MYOD/MYOG Expression Targets |
|  | IGF1 -> STAT Expression Targets |
|  | IGF1 -> ELK/SRF/HIF1A/MYC/SREBF Expression Targets |
|  | Insulin -> STAT Expression Targets |
|  | Insulin -> CEBPA/CTNNB/FOXA/FOXO Expression Targets |
|  | Insulin -> MEF/MYOD Expression Targets |
|  | Insulin -> ELK/SRF/HIF1A/MYC/SREBF Expression Targets |
|  | Fibrinogen Expression Targets |
|  | EGFR -> Expression Targets in Skin |
|  | IL3 Expression Targets |
|  | Leptin -> STAT Expression Targets |
|  | Leptin -> ELK/SRF Expression Targets |
|  | Prostaglandin F Expression Targets |
|  | LPA Expression Targets |
|  | PAF/Gq -> AP-1/ATF1/CREB/ERK/SRF Expression Targets |
|  | Dronabinol/Anandamide Expression Targets |
|  | Morphine Expression Targets |
|  | S1P Expression Targets |
|  | EGFR-> Expression Targets in Lymphoid System and Blood |
|  | GPCRs Family -> Expression Targets in Lymphoid System and Blood |
|  | NRG1 -> MEF/MYOD Expression Targets |
|  | NRG1 -> CREB/CREBBP/ELK/SRF/MYC Expression Targets |
|  | NRG1 -> FOXO3A Expression Targets |
|  | NRG1 -> TP53 Expression Target |
|  | NRG1/Catenin Expression Targets |
|  | NRG1 -> STAT Expression Targets |
|  | NRG1 -> AP-1/ATF Expression Targets |
|  | NRG1 -> HIF1A Expression Target |
|  | NOTCH 1/3 -> Expression Targets in Nerve Tissue |
|  | Elevated Receptors -> Expression Targets in Nerve Tissue |
|  | Elevated Receptors -> Expression Targets in Parathyroid Gland |
|  | NGF -> SMAD3/NF-kB Expression Targets |
|  | NTF3 Expression Targets |
|  | DLL4 Expression Targets |
|  | ADAM17 Expression Targets |
|  | DLL1 Expression Targets |
|  | NOTCH Expression Targets |
|  | PDGF -> AP-1/CREB/CREBBP/MYC Expression Targets |
|  | PDGF -> STAT Expression Targets |
|  | AVP/Gs -> CREB/ELK/SRF/AP-1/EGR Expression Targets |
|  | GNRH1 Expression Targets |
|  | NTS Expression Targets |
|  | EDN1 Expression Targets |
|  | AGT -> CREB Expression Targets |
|  | AVP/Gs -> MEF/MYOD/NFATC/MYOG Expression Targets |
|  | AVP/Gq -> STAT Expression Targets |
|  | IFNA1/Gq Expression Targets |
|  | AGT -> ELK/SRF Expression Targets |
|  | TAC1 Expression Targets |
|  | AVP/Gs -> STAT Expression Targets |
|  | AVP/Gq -> MEF/MYOD/NFATC/MYOG Expression Targets |
|  | AGT -> STAT Expression Targets |
|  | AVP/Gq -> CREB/ELK/SRF/AP-1/EGR Expression Targets |
|  | KITLG -> STAT Expression Targets |
|  | KITLG -> AP-1/CREB/CREBBP/MYC Expression Targets |
|  | PAF Expression Targets |
|  | Elevated Receptors -> Expression Targets in Breast |
|  | Elevated Receptors -> Expression Targets in Endometrium |
|  | Elevated Receptors -> Expression Targets in Placenta |
|  | Elevated Receptors -> Expression Targets in Respiratory System |
|  | FOXO1 Signaling |
|  | Estrogens/ESR1 non-Genomic Signaling |
|  | EGFR Signaling |
|  | HRAS Signaling |
|  | ERK/MAPK Canonical Signaling |
|  | TGFA -> STAT Expression Targets |
|  | TGFA -> FOXO3A Expression Targets |
|  | TGFA -> CTNNB/CTNND Expression Targets |
|  | TGFA -> TP53 Expression Targets |
|  | TGFA -> HIF1A Expression Targets |
|  | TGFA -> MEF/MYOD/NFATC Expression Targets |
|  | TGFA/AP-1/ATF Expression Targets |
|  | TGFA -> CREB/CREBBP/ELK-SRF/MYC Expression Targets |
|  | TGFB1-TGFBR2 Expression Targets |
|  | TGFB1-TGFBR1/AP-1 Expression Targets |
|  | TGFB1-ACVRL1 Expression Targets |
|  | TNF -> NF-kB Expression Targets |
|  | TNF -> AP-1 Expression Targets |
|  | TNF -> TP53/ATF Expression Targets |
|  | EDA Expression Targets |
|  | IL1B -> PGE2 Expression Targets |
|  | IL1B Expression Targets |
|  | IL1A Expression Targets |
|  | ROS in Angiotensin Mediated Cardiovascular Remodeling and Hypertrophy |
|  | WNT1 Expression Targets |
|  | WNT7B Expression Targets |
|  | WNT5A Expression Targets |
|  | ace2 |
|  | prarg negative regulators, ps-positive |
|  | pprarg neg, uknown targets, ps-positive |
|  | prarg neg,ukn expres targets, ps-positive |
|  | PPARG negative regulators |
|  | PPARG negative targets |
|  | pparg expr target\_regulators |
|  | Neighbors of disease exacerbation |
|  | Neighbors of tumor progression |
|  | Neighbors of cancer progression |
|  | Neighbors of inflammatory status |
|  | Neighbors of overall survival |
|  | Neighbors of disease severity |
|  | Neighbors of tumor size |
|  | Neighbors of lesion size |
|  | Neighbors of mortality |
|  | Neighbors of survival rate |
|  | Neighbors of infarct size |
|  | Neighbors of survival time |
|  | Neighbors of functional recovery |
|  | Neighbors of insulin sensitivity |
|  | Neighbors of bacterial load |
|  | Neighbors of cancer survival |
|  | Neighbors of graft survival |
|  | Neighbors of therapeutic efficacy |
|  | Neighbors of clinical stage |
|  | Neighbors of lethality |
|  | Neighbors of tumor regression |
|  | Neighbors of recurrence-free survival |
|  | Neighbors of disease-free survival |
|  | Neighbors of tumor response |
|  | Neighbors of macrophage count |
|  | Neighbors of long-term survival |
|  | Neighbors of treatment outcome |
|  | Neighbors of bone mass |
|  | Neighbors of body weight |
|  | Neighbors of body weight gain |
|  | Neighbors of cardiovascular risk |
|  | Neighbors of microvessel density |
|  | Neighbors of tumor recurrence |
|  | Neighbors of tumor promotion |
|  | Neighbors of neutrophil count |
|  | Neighbors of glucose tolerance |
|  | Neighbors of morbidity |
|  | Neighbors of fibrosis progression |
|  | Neighbors of mortality rate |
|  | Neighbors of adverse outcome |
|  | Neighbors of radioresistance |
|  | Neighbors of cell density |
|  | Neighbors of cancer-specific survival |
|  | Neighbors of progression-free survival |
|  | Neighbors of apoptosis rate |
|  | Neighbors of shear stress |
|  | Neighbors of viral load |
|  | Neighbors of blood pressure |
|  | Neighbors of leukocyte count |
|  | Neighbors of tumor grade |
|  | Neighbors of vascular reactivity |
|  | Neighbors of vascular leakage |
|  | Neighbors of vascular density |
|  | Neighbors of muscle mass |
|  | Neighbors of cancer risk |
|  | Neighbors of cancer incidence |
|  | Neighbors of tumor aggressiveness |
|  | Neighbors of systolic blood pressure |
|  | Neighbors of tumor resistance |
|  | Neighbors of blood glucose |
|  | Neighbors of ejection fraction |
|  | Neighbors of median survival |
|  | Neighbors of renal hypertrophy |
|  | Neighbors of fasting plasma glucose |
|  | Neighbors of osteoclast number |
|  | Neighbors of T-cell count |
|  | Neighbors of antimetastatic activity |
|  | Neighbors of energy expenditure |
|  | Neighbors of glucose level |
|  | Neighbors of vascular tone |
|  | Neighbors of radiosensitivity |
|  | Neighbors of left ventricular ejection fraction |
|  | Neighbors of breast cancer risk |
|  | Neighbors of proliferation index |
|  | Neighbors of fertility |
|  | Neighbors of recurrence rate |
|  | Neighbors of pathological response |
|  | Neighbors of skin thickness |
|  | Neighbors of metabolic response |
|  | Neighbors of insulin level |
|  | Neighbors of comorbidity |
|  | Neighbors of disease exacerbation |
|  | Neighbors of tumor progression |
|  | Neighbors of cancer progression |
|  | Neighbors of inflammatory status |
|  | Neighbors of overall survival |
|  | Neighbors of disease severity |
|  | Neighbors of tumor size |
|  | Neighbors of lesion size |
|  | Neighbors of mortality |
|  | Neighbors of survival rate |
|  | Neighbors of infarct size |
|  | Neighbors of survival time |
|  | Neighbors of functional recovery |
|  | Neighbors of insulin sensitivity |
|  | Neighbors of bacterial load |
|  | Neighbors of cancer survival |
|  | Neighbors of graft survival |
|  | Neighbors of therapeutic efficacy |
|  | Neighbors of clinical stage |
|  | Neighbors of lethality |
|  | Neighbors of tumor regression |
|  | Neighbors of recurrence-free survival |
|  | Neighbors of disease-free survival |
|  | Neighbors of tumor response |
|  | Neighbors of macrophage count |
|  | Neighbors of long-term survival |
|  | Neighbors of treatment outcome |
|  | Neighbors of bone mass |
|  | Neighbors of body weight |
|  | Neighbors of body weight gain |
|  | Neighbors of cardiovascular risk |
|  | Neighbors of microvessel density |
|  | Neighbors of tumor recurrence |
|  | Neighbors of tumor promotion |
|  | Neighbors of neutrophil count |
|  | Neighbors of glucose tolerance |
|  | Neighbors of morbidity |
|  | Neighbors of fibrosis progression |
|  | Neighbors of mortality rate |
|  | Neighbors of adverse outcome |
|  | Neighbors of radioresistance |
|  | Neighbors of cell density |
|  | Neighbors of cancer-specific survival |
|  | Neighbors of progression-free survival |
|  | Neighbors of apoptosis rate |
|  | Neighbors of shear stress |
|  | Neighbors of viral load |
|  | Neighbors of blood pressure |
|  | Neighbors of leukocyte count |
|  | Neighbors of tumor grade |
|  | Neighbors of vascular reactivity |
|  | Neighbors of vascular leakage |
|  | Neighbors of vascular density |
|  | Neighbors of muscle mass |
|  | Neighbors of cancer risk |
|  | Neighbors of cancer incidence |
|  | Neighbors of tumor aggressiveness |
|  | Neighbors of systolic blood pressure |
|  | Neighbors of tumor resistance |
|  | Neighbors of blood glucose |
|  | Neighbors of ejection fraction |
|  | Neighbors of median survival |
|  | Neighbors of renal hypertrophy |
|  | Neighbors of fasting plasma glucose |
|  | Neighbors of osteoclast number |
|  | Neighbors of T-cell count |
|  | Neighbors of antimetastatic activity |
|  | Neighbors of energy expenditure |
|  | Neighbors of glucose level |
|  | Neighbors of vascular tone |
|  | Neighbors of radiosensitivity |
|  | Neighbors of left ventricular ejection fraction |
|  | Neighbors of breast cancer risk |
|  | Neighbors of proliferation index |
|  | Neighbors of fertility |
|  | Neighbors of recurrence rate |
|  | Neighbors of pathological response |
|  | Neighbors of skin thickness |
|  | Neighbors of metabolic response |
|  | Neighbors of insulin level |
|  | Neighbors of comorbidity |
|  | Neighbors of cell development |
|  | Neighbors of immune response |
|  | Neighbors of cell population |
|  | Neighbors of T-cell development |
|  | Neighbors of inflammatory response |
|  | Neighbors of wound healing |
|  | Neighbors of angiogenesis |
|  | Neighbors of innate immune response |
|  | Neighbors of cell function |
|  | Neighbors of macrophage activation |
|  | Neighbors of cell formation |
|  | Neighbors of adaptive immune response |
|  | Neighbors of osteoclast development |
|  | Neighbors of epithelial to mesenchymal transition |
|  | Neighbors of immunity |
|  | Neighbors of cell proliferative response |
|  | Neighbors of chemotaxis |
|  | Neighbors of dendritic cell differentiation |
|  | Neighbors of cell survival |
|  | Neighbors of osteoclast differentiation |
|  | Neighbors of cell phenotype |
|  | Neighbors of T-cell proliferation |
|  | Neighbors of tissue remodeling |
|  | Neighbors of cell adhesion |
|  | Neighbors of T-cell activation |
|  | Neighbors of endothelial cell proliferation |
|  | Neighbors of fibrogenesis |
|  | Neighbors of tumor growth |
|  | Neighbors of dendritic cell development |
|  | Neighbors of tissue repair |
|  | Neighbors of cell count |
|  | Neighbors of cell infiltration |
|  | Neighbors of cell interaction |
|  | Neighbors of SMC proliferation |
|  | Neighbors of lumen formation |
|  | Neighbors of endothelial cell migration |
|  | Neighbors of adipogenesis |
|  | Neighbors of lipid storage |
|  | Neighbors of cell damage |
|  | Neighbors of pregnancy |
|  | Neighbors of aging |
|  | Neighbors of cell motility |
|  | Neighbors of bone resorption |
|  | Neighbors of fibroblast proliferation |
|  | Neighbors of adipocyte differentiation |
|  | Neighbors of macrophage migration |
|  | Neighbors of microglial activation |
|  | Neighbors of neutrophil recruitment |
|  | Neighbors of regeneration |
|  | Neighbors of cell migration |
|  | Neighbors of cancer growth |
|  | Neighbors of stem cell proliferation |
|  | Neighbors of smooth muscle cell migration |
|  | Neighbors of hepatic regeneration |
|  | Neighbors of transendothelial migration |
|  | Neighbors of phagocytosis |
|  | Neighbors of immune system activation |
|  | Neighbors of leukocyte migration |
|  | Neighbors of leukocyte recruitment |
|  | Neighbors of osteoclast formation |
|  | Neighbors of monocyte recruitment |
|  | Neighbors of cell invasion |
|  | Neighbors of heart function |
|  | Neighbors of hemopoiesis |
|  | Neighbors of ossification |
|  | Neighbors of T-cell function |
|  | Neighbors of immune system function |
|  | Neighbors of lung development |
|  | Neighbors of autophagy |
|  | Neighbors of monocyte adhesion |
|  | Neighbors of transcription activation |
|  | Neighbors of senescence |
|  | Neighbors of T-cell response |
|  | Neighbors of tumor immunity |
|  | Neighbors of hepatocyte proliferation |
|  | Neighbors of cell transdifferentiation |
|  | Neighbors of neuroprotection |
|  | Neighbors of stem cell differentiation |
|  | Neighbors of endothelial cell function |
|  | Neighbors of neuronal death |
|  | Neighbors of cell growth |
|  | Neighbors of ROS generation |
|  | Neighbors of epithelial cell proliferation |
|  | Neighbors of first trimester pregnancy |
|  | Neighbors of cancer cell growth |
|  | Neighbors of sensitization |
|  | Neighbors of hemato-encephalic barrier |
|  | Neighbors of macrophage function |
|  | Neighbors of cellular immune response |
|  | Neighbors of cellular senescence |
|  | Neighbors of colony formation |
|  | Neighbors of cell differentiation |
|  | Neighbors of keratinocyte proliferation |
|  | Neighbors of stem cell migration |
|  | Neighbors of life span |
|  | Neighbors of cell homeostasis |
|  | Neighbors of psoriasis |
|  | Neighbors of inflammatory disease |
|  | Neighbors of rheumatoid arthritis |
|  | Neighbors of atherosclerosis |
|  | Neighbors of fibrosis |
|  | Neighbors of colitis |
|  | Neighbors of arthritis |
|  | Neighbors of inflammatory bowel disease |
|  | Neighbors of injury |
|  | Neighbors of atherogenesis |
|  | Neighbors of vascular remodeling |
|  | Neighbors of metastasis |
|  | Neighbors of carcinogenesis |
|  | Neighbors of multiple sclerosis |
|  | Neighbors of chronic inflammation |
|  | Neighbors of inflammation |
|  | Neighbors of tumor microenvironment |
|  | Neighbors of pathological angiogenesis |
|  | Neighbors of obesity |
|  | Neighbors of vascular disease |
|  | Neighbors of diabetes mellitus |
|  | Neighbors of asthma |
|  | Neighbors of pneumonia |
|  | Neighbors of hyperplasia |
|  | Neighbors of osteoarthritis |
|  | Neighbors of liver fibrosis |
|  | Neighbors of chronic obstructive pulmonary disease |
|  | Neighbors of myocardial infarction |
|  | Neighbors of leukocyte infiltration |
|  | Neighbors of reperfusion injury |
|  | Neighbors of airway inflammation |
|  | Neighbors of insulin resistance |
|  | Neighbors of infection |
|  | Neighbors of vascular injury |
|  | Neighbors of renal injury |
|  | Neighbors of preeclampsia |
|  | Neighbors of macrophage infiltration |
|  | Neighbors of liver injury |
|  | Neighbors of neuroinflammation |
|  | Neighbors of death |
|  | Neighbors of type 1 diabetes |
|  | Neighbors of dermatitis |
|  | Neighbors of neutrophil infiltration |
|  | Neighbors of heart disease |
|  | Neighbors of diabetic nephropathy |
|  | Neighbors of lung injury |
|  | Neighbors of systemic sclerosis |
|  | Neighbors of hepatocellular carcinoma |
|  | Neighbors of acute lung injury |
|  | Neighbors of gastric cancer |
|  | Neighbors of colorectal cancer |
|  | Neighbors of cardiac remodeling |
|  | Neighbors of breast cancer |
|  | Neighbors of ischemia |
|  | Neighbors of systemic lupus erythematosus |
|  | Neighbors of melanoma |
|  | Neighbors of collagen-induced arthritis |
|  | Neighbors of cartilage degeneration |
|  | Neighbors of interstitial fibrosis |
|  | Neighbors of neoplasm invasion |
|  | Neighbors of kidney disease |
|  | Neighbors of heart failure |
|  | Neighbors of lung disease |
|  | Neighbors of pulmonary fibrosis |
|  | Neighbors of cardiovascular disease |
|  | Neighbors of stroke |
|  | Neighbors of cancer |
|  | Neighbors of type 2 diabetes |
|  | Neighbors of neoplasm |
|  | Neighbors of endometriosis |
|  | Neighbors of neointima |
|  | Neighbors of atrophy |
|  | Neighbors of diet-induced obesity |
|  | Neighbors of respiratory hypersensitivity |
|  | Neighbors of myocardial fibrosis |
|  | Neighbors of synovitis |
|  | Neighbors of rupture |
|  | Neighbors of endothelial cell dysfunction |
|  | Neighbors of prostate cancer |
|  | Neighbors of hypertrophy |
|  | Neighbors of osteolysis |
|  | Neighbors of ovarian cancer |
|  | Neighbors of pancreatic cancer |
|  | Neighbors of hypertension |
|  | Neighbors of intimal hyperplasia |
|  | Neighbors of proteinuria |
|  | Neighbors of epithelial cell |
|  | Neighbors of keratinocyte |
|  | Neighbors of monocyte |
|  | Neighbors of macrophage |
|  | Neighbors of fibroblast |
|  | Neighbors of airway epithelial cell |
|  | Neighbors of stromal cell |
|  | Neighbors of osteoblast |
|  | Neighbors of pneumocyte |
|  | Neighbors of intestine epithelium cell |
|  | Neighbors of mononuclear cell |
|  | Neighbors of hepatocyte |
|  | Neighbors of neutrophil |
|  | Neighbors of astrocyte |
|  | Neighbors of stem cell |
|  | Neighbors of granulosa cell |
|  | Neighbors of T-cell |
|  | Neighbors of neuroglia |
|  | Neighbors of lung |
|  | Neighbors of liver |
|  | Neighbors of kidney |
|  | Neighbors of skin |
|  | Neighbors of intestine |
|  | Neighbors of lymph node |
|  | Neighbors of colon |
|  | Neighbors of airway |
|  | Neighbors of blood vessel |
|  | Neighbors of heart |
|  | Neighbors of gastrointestinal tract |
|  | Neighbors of bone |
|  | Neighbors of placenta |
|  | Neighbors of esophagus |
|  | Neighbors of brain |
|  | Neighbors of ovary |
|  | Neighbors of breast |
|  | Neighbors of stomach |
|  | Neighbors of pancreas |
|  | Neighbors of prostate |
|  | Neighbors of central nervous system |
|  | Neighbors of colorectal region |
|  | Neighbors of alveolus |
|  | Neighbors of vein |
|  | Neighbors of eye |
|  | Neighbors of lymph node of mesentery |
|  | Neighbors of mammary gland |
|  | Neighbors of cerebral cortex |
|  | Neighbors of bladder |
|  | Neighbors of nervous system |
|  | Neighbors of blastocyst |
|  | Neighbors of endocrine system |
|  | Neighbors of lymphatic vessels |
|  | Neighbors of pleura |
|  | Neighbors of hair follicle |
|  | Neighbors of NF-kB family |
|  | Neighbors of oxidized LDL |
|  | Neighbors of JNK |
|  | Neighbors of PPAR |
|  | Neighbors of Jun/Fos |
|  | Neighbors of PKC |
|  | Neighbors of PI3K |
|  | Neighbors of mitogen-activated protein kinase |
|  | Neighbors of IFNAR ligand |
|  | Neighbors of transforming growth factor |
|  | Neighbors of cytokine |
|  | Neighbors of interferon |
|  | Neighbors of PDGF |
|  | Neighbors of Ras GTPase |
|  | Neighbors of ERK1/2 |
|  | Neighbors of growth factor |
|  | Neighbors of PKA |
|  | Neighbors of ovalbumin |
|  | Neighbors of LDL |
|  | Neighbors of NF-AT family |
|  | Neighbors of beta adrenoceptor |
|  | Neighbors of chorionic gonadotropin |
|  | Neighbors of IKBK |
|  | Neighbors of protein tyrosine kinase |
|  | Neighbors of NAD(P)H oxidase |
|  | Neighbors of IgG |
|  | Neighbors of SMAD subfamily |
|  | Neighbors of AMPK |
|  | Neighbors of estrogen receptor |
|  | Neighbors of histone deacetylase |
|  | Neighbors of PDGF-BB |
|  | Neighbors of IL1R |
|  | Neighbors of HIF-1 |
|  | Neighbors of GPCR |
|  | Neighbors of Notch |
|  | Neighbors of heat shock protein 90 |
|  | Neighbors of lectin |
|  | Neighbors of fibrinogen |
|  | Neighbors of endothelin |
|  | Neighbors of NOS |
|  | Neighbors of proteasome endopeptidase complex |
|  | Neighbors of class nuclear receptor with C4 zinc fingers |
|  | Neighbors of non-coding RNA |
|  | Neighbors of histone H3 |
|  | Neighbors of alpha2 adrenoceptor |
|  | Neighbors of vitronectin receptor (integrin) |
|  | Neighbors of retinoid-X receptor subfamily |
|  | Neighbors of WNT |
|  | Neighbors of gonadotropins |
|  | Neighbors of immunoglobulin |
|  | Neighbors of phospholipase |
|  | Neighbors of ITG |
|  | Neighbors of FSH |
|  | Neighbors of RA receptor |
|  | Neighbors of neuropeptides |
|  | Neighbors of adrenoceptor |
|  | Neighbors of caspase |
|  | Neighbors of phosphoinositide phospholipase C |
|  | Neighbors of TNF-alpha receptor |
|  | Model of PPARG signaling in psoriasis |
|  | PPARG negative regulators and targets |
|  | Model of PPARG related pathways in psoriasis (short version) |
|  | 3\_Mesangial cell dysfunction in glomerulonephritis |
|  | 2\_Pathogens stimulate mucins expression in the middle ear |
|  | 2\_The intense proliferation and contractile activity of smooth muscle cells in pulmonary hypertension |
|  | 3\_1\_Mucus accumulation in asthma |
|  | 4\_Mucin hyperproduction in goblet and mucous cells in COPD |
|  | 3\_Human papilloma virus E4 and E5 proteins in viral reproduction |
|  | drug-target pairs binding |
|  | drug-target pairs direct regulation |
|  | New Combined Pathway |
|  | Model of PPARG signaling in psoriais (tested) |
|  | opcs |
|  | DIPG cell biomarkers |
|  | mapk1 protein modificators |

---

|  |  |
| --- | --- |
| Group | Genes with Mutations Associated with Colorectal Cancer |
|  | Genes with Mutations Associated with Primary Glioblastoma |
|  | Genes with Mutations Associated with Glioma |
|  | Chemokines, Growth Factors and Receptors Involved in Cancer Cells Chemotaxis |

---

|  |  |
| --- | --- |
| MedScan ID | 1956 |

---

|  |  |
| --- | --- |
| LocusLink ID | 1956 |
|  | 13649 |
|  | 24329 |

---

|  |  |
| --- | --- |
| Alias | RP23-295E4.1 |
|  | erbB receptor |
|  | avian erythroblastic leukemia viral (v-erb-b) oncogene homolog |
|  | v-erbB oncogene homolog |
|  | v-erb-b |
|  | epidermal growth factor receptor 1 |
|  | HER1 |
|  | EGF-Rs |
|  | transforming growth factor-alpha receptor |
|  | TGF-alpha receptor |
|  | epidermal growth factor receptor vIII |
|  | epidermal growth factor receptor variant III |
|  | Egfr |
|  | EGF-R |
|  | truncated epidermal growth factor receptor |
|  | EGFR-related peptide |
|  | cell proliferation-inducing protein 61 |
|  | Errb1 |
|  | epidermal growth factor receptor (erythroblastic leukemia viral (v-erb-b) oncogene homolog, avian) |
|  | epidermal growth factor receptors |
|  | avian erythroblastic leukemia viral (v-erbB) oncogene homolog |
|  | Epidermal growth factor receptor formerly avian erythroblastic leukemia viral (v-erbB) oncogene homolog |
|  | 9030024J15 |
|  | Errp |
|  | GNB:6478868 |
|  | EGF receptors |
|  | OTTMUSP00000005385 |
|  | epidermal growth factor receptor |
|  | erythroblastic leukemia viral (v-erb-b) oncogene homolog |
|  | erb-b2 receptor tyrosine kinase 1 |
|  | mENA |
|  | receptor tyrosine-protein kinase erbB-1 |
|  | EGFR\_HUMAN |
|  | EGFRvIII |
|  | PIG61 |
|  | AI552599 |
|  | OTTHUMP00000209210 |
|  | cell growth inhibiting protein 40 |
|  | OTTMUSP00000005386 |
|  | RIKEN cDNA 9030024J15 gene |
|  | EGF receptor |
|  | ERBB I |
|  | erythroblastic leukemia viral (v-erb-b) oncogene homolog, avian |
|  | v-erb-bs |
|  | EGF/TGF-alpha receptor |
|  | TGF-alpha receptors |
|  | EGFR1 |
|  | NISBD2 |
|  | LOC360274 |
|  | ERB1 |
|  | Epidermal growth factor receptor formerly avian erythroblastic leukemia viral (v-erbB) oncogene homolog (Erbb1) |
|  | proto-oncogene c-ErbB-1 |
|  | EGFR\_MOUSE |
|  | receptor for epidermal growth factor |
|  | EGFRs |
|  | ERBB |
|  | wa II |
|  | ERBB1 |
|  | transforming growth factor-alpha receptors |
|  | erbB receptors |
|  | EGF holoreceptor |
|  | EGFR vIII |
|  | 9030024J15Rik |
|  | waved 2 |
|  | epidermal growth factor receptor tyrosine kinase domain |
|  | Wa5 |
|  | wa2 |
|  | wa-2 |
|  | ErbB-1 |
|  | epidermal growth factor receptor, formerly avian erythroblastic leukemia viral (v-erbB) oncogene homolog (Erbb1) |
|  | erythroblastic leukaemia viral (v-erb-b) oncogene homolog, avian |
|  | epidermal growth factor receptor (erythroblastic leukaemia viral (v-erb-b) oncogene homolog, avian) |
|  | avian erythroblastic leukaemia viral (v-erb-b) oncogene homolog |
|  | avian erythroblastic leukaemia viral (v-erbB) oncogene homolog |
|  | Epidermal growth factor receptor formerly avian erythroblastic leukaemia viral (v-erbB) oncogene homolog |
|  | erythroblastic leukaemia viral (v-erb-b) oncogene homolog |
|  | epidermal growth factor receptor (avian erythroblastic leukemia viral (v-er |
|  | epidermal growth factor receptor (avian erythroblastic leukaemia viral (v-er |

---

|  |  |
| --- | --- |
| EC Number | 2.7.10.1 |

---

|  |  |
| --- | --- |
| GO ID | 0005524 |
|  | 0004709 |
|  | 0051015 |
|  | 0045296 |
|  | 0005516 |
|  | 0003682 |
|  | 0003690 |
|  | 0019899 |
|  | 0048408 |
|  | 0005006 |
|  | 0005178 |
|  | 0030235 |
|  | 0046982 |
|  | 0019901 |
|  | 0019903 |
|  | 0004713 |
|  | 0004714 |
|  | 0004888 |
|  | 0031625 |
|  | 0001618 |
|  | 0038128 |
|  | 0000165 |
|  | 0000186 |
|  | 0043006 |
|  | 0007202 |
|  | 0048143 |
|  | 0030154 |
|  | 0008283 |
|  | 0007166 |
|  | 0098609 |
|  | 0071230 |
|  | 0071276 |
|  | 0071549 |
|  | 0071364 |
|  | 0071392 |
|  | 0071260 |
|  | 0034614 |
|  | 0021795 |
|  | 0007623 |
|  | 0048546 |
|  | 0016101 |
|  | 0001892 |
|  | 0007173 |
|  | 0061029 |
|  | 0001942 |
|  | 0042743 |
|  | 0007611 |
|  | 0097421 |
|  | 0030324 |
|  | 0010960 |
|  | 0061024 |
|  | 0007494 |
|  | 0060571 |
|  | 1901185 |
|  | 0045746 |
|  | 0043066 |
|  | 1905208 |
|  | 0042059 |
|  | 0045930 |
|  | 0042177 |
|  | 0048812 |
|  | 0001503 |
|  | 0042698 |
|  | 0038083 |
|  | 0018108 |
|  | 0045739 |
|  | 0045740 |
|  | 0070374 |
|  | 1900087 |
|  | 0043406 |
|  | 1901224 |
|  | 0097755 |
|  | 0045780 |
|  | 0090263 |
|  | 0030307 |
|  | 0030335 |
|  | 0008284 |
|  | 0045737 |
|  | 0050679 |
|  | 0048146 |
|  | 0050729 |
|  | 0010750 |
|  | 0033138 |
|  | 0042327 |
|  | 1903800 |
|  | 1902722 |
|  | 0051897 |
|  | 1900020 |
|  | 1903078 |
|  | 0001934 |
|  | 0048661 |
|  | 0032930 |
|  | 0051968 |
|  | 0045944 |
|  | 0045893 |
|  | 0045907 |
|  | 0046777 |
|  | 0051205 |
|  | 0070372 |
|  | 0046328 |
|  | 2000145 |
|  | 0050999 |
|  | 0050730 |
|  | 0014066 |
|  | 0006357 |
|  | 0070141 |
|  | 0051592 |
|  | 0033590 |
|  | 0033594 |
|  | 0006970 |
|  | 0007435 |
|  | 0007165 |
|  | 0043586 |
|  | 0006412 |
|  | 0007169 |
|  | 0046718 |
|  | 0042060 |
|  | 0000139 |
|  | 0070435 |
|  | 0016324 |
|  | 0009925 |
|  | 0016323 |
|  | 0009986 |
|  | 0030665 |
|  | 0005737 |
|  | 0031901 |
|  | 0030139 |
|  | 0005789 |
|  | 0005768 |
|  | 0010008 |
|  | 0005615 |
|  | 0005925 |
|  | 0005887 |
|  | 0016020 |
|  | 0045121 |
|  | 0097489 |
|  | 0031965 |
|  | 0005634 |
|  | 0048471 |
|  | 0005886 |
|  | 0032991 |
|  | 0043235 |
|  | 0045202 |
|  | 0016301 |
|  | 0000166 |
|  | 0004672 |
|  | 0005102 |
|  | 0016740 |
|  | 0000902 |
|  | 0071363 |
|  | 0008544 |
|  | 0035556 |
|  | 0007275 |
|  | 0016310 |
|  | 0006468 |
|  | 0006898 |
|  | 0042127 |
|  | 0005794 |
|  | 0005783 |
|  | 0016021 |
|  | 0035690 |
|  | 0001889 |
|  | 0032355 |
|  | 0033993 |
|  | 0014070 |
|  | 0006979 |
|  | 0005088 |
|  | 0046934 |
|  | 0004716 |
|  | 0046854 |
|  | 0048015 |
|  | 0043547 |
|  | 0035413 |
|  | 0031659 |
|  | 0045429 |
|  | 0006950 |
|  | 0016337 |
|  | 0030122 |
|  | 0001948 |
|  | 0004871 |
|  | 0045909 |
|  | 0005622 |
|  | 0023014 |
|  | 0038095 |
|  | 0019694 |
|  | 0007411 |
|  | 0008543 |
|  | 0045087 |
|  | 0048011 |
|  | 0016772 |
|  | 0005976 |

---

|  |  |
| --- | --- |
| KEGG ID | hsa:1956 |
|  | mmu:13649 |
|  | rno:24329 |

---

|  |  |
| --- | --- |
| Organism | Homo sapiens {Organism urn:agi-taxid:9606} |
|  | Mus musculus {Organism urn:agi-taxid:10090} |
|  | Rattus norvegicus {Organism urn:agi-taxid:10116} |
|  | Homo sapiens |
|  | Mus musculus |
|  | Rattus norvegicus |

---

|  |  |
| --- | --- |
| Mouse chromosome position | 11 9.41 cM |

---

|  |  |
| --- | --- |
| OMIM ID | 131550 |
|  | 616069 |
|  | 211980 |

---

|  |  |
| --- | --- |
| Rat chromosome position | 14q22 |

---

|  |  |
| --- | --- |
| Hugo ID | 3236 |
|  | HGNC:3236 |

---

|  |  |
| --- | --- |
| Human chromosome position | 7p11.2 |
|  | 7p12 |

---

|  |  |
| --- | --- |
| Swiss-Prot Accession | Q504U8 |
|  | E7BSV0 |
|  | F2YGG7 |
|  | E9PFD7 |
|  | B7Z2I3 |
|  | P00533 |
|  | P00533.2 |
|  | Q9WVF5 |
|  | Q01279 |
|  | Q3TQS6 |
|  | Q9EP98 |
|  | Q01279.1 |
|  | Q9QX70 |
|  | O00688 |
|  | O00732 |
|  | P06268 |
|  | Q14225 |
|  | Q68GS5 |
|  | Q92795 |
|  | Q9BZS2 |
|  | Q9GZX1 |
|  | Q9H2C9 |
|  | Q9H3C9 |
|  | Q9UMD7 |
|  | Q9UMD8 |
|  | Q9UMG5 |

---

|  |  |
| --- | --- |
| PIR ID | A00641 |
|  | A53183 |

---

|  |  |
| --- | --- |
| GenBank ID | NC\_000007 |
|  | NM\_005228 |
|  | NP\_005219 |
|  | NM\_001346941 |
|  | NP\_001333870 |
|  | NM\_001346898 |
|  | NP\_001333827 |
|  | NM\_001346897 |
|  | NP\_001333826 |
|  | NM\_201284 |
|  | NP\_958441 |
|  | NM\_201282 |
|  | NP\_958439 |
|  | NM\_001346899 |
|  | NP\_001333828 |
|  | NM\_201283 |
|  | NP\_958440 |
|  | NM\_001346900 |
|  | NP\_001333829 |
|  | NG\_007726 |
|  | AB221348 |
|  | BAE15958 |
|  | AB360371 |
|  | BAF79636 |
|  | AB360855 |
|  | BAF80003 |
|  | AB360856 |
|  | BAF80004 |
|  | AB685813 |
|  | BAL40868 |
|  | AB686642 |
|  | BAL41217 |
|  | AC006977 |
|  | AC073324 |
|  | AAS07524 |
|  | AF040717 |
|  | AF288738 |
|  | AAG35786 |
|  | AAG35787 |
|  | AAG35788 |
|  | AAG35789 |
|  | AAG35790 |
|  | AH006650 |
|  | AAC50796 |
|  | AAC50797 |
|  | AAC50798 |
|  | AAC50799 |
|  | AAC50800 |
|  | AAC50801 |
|  | AH010139 |
|  | AAG43243 |
|  | AAG43244 |
|  | AAG43245 |
|  | AY588246 |
|  | AAS83109 |
|  | CH236957 |
|  | CH471201 |
|  | EAW50962 |
|  | EAW50963 |
|  | EAW50964 |
|  | EAW50965 |
|  | CS238505 |
|  | CAJ55813 |
|  | CS542488 |
|  | CAM91181 |
|  | GM619915 |
|  | CAV33298 |
|  | HC464560 |
|  | CBK51921 |
|  | HC464562 |
|  | CBK51922 |
|  | HC464564 |
|  | CBK51923 |
|  | HC464566 |
|  | CBK51924 |
|  | HF548011 |
|  | CCO13722 |
|  | J03206 |
|  | JA807020 |
|  | CCO75437 |
|  | JA807083 |
|  | CCO75439 |
|  | JA913745 |
|  | CCV20014 |
|  | JC478129 |
|  | CEF34368 |
|  | JQ739160 |
|  | AFK93471 |
|  | JQ739161 |
|  | AFK93472 |
|  | JQ739162 |
|  | AFK93473 |
|  | JQ739163 |
|  | AFK93474 |
|  | JQ739164 |
|  | AFK93475 |
|  | JQ739165 |
|  | AFK93476 |
|  | JQ739166 |
|  | AFK93477 |
|  | JQ739167 |
|  | AFK93478 |
|  | KF458469 |
|  | KJ685773 |
|  | AIE16170 |
|  | KJ685774 |
|  | AIE16171 |
|  | KT584716 |
|  | KX162725 |
|  | ART34939 |
|  | KX162726 |
|  | ART34940 |
|  | KX162727 |
|  | ART34941 |
|  | KX162728 |
|  | ART34942 |
|  | KX162729 |
|  | ART34943 |
|  | KX162730 |
|  | ART34944 |
|  | KX162731 |
|  | ART34945 |
|  | KX819315 |
|  | ART92273 |
|  | M11234 |
|  | AAA52370 |
|  | M23390 |
|  | M38425 |
|  | AAA63171 |
|  | U48723 |
|  | AAC50803 |
|  | AAC50804 |
|  | X06370 |
|  | CAA29668 |
|  | X17054 |
|  | CAA34902 |
|  | AB209442 |
|  | BAD92679 |
|  | AF125253 |
|  | AAG43240 |
|  | AF277897 |
|  | AAK01080 |
|  | AI217671 |
|  | AK127817 |
|  | AK225422 |
|  | AK290352 |
|  | BAF83041 |
|  | AK294750 |
|  | BAH11869 |
|  | AL598260 |
|  | AU137334 |
|  | AW163038 |
|  | AW295229 |
|  | AW450657 |
|  | AY550966 |
|  | AAT52212 |
|  | AY698023 |
|  | AAT97978 |
|  | AY698024 |
|  | AAT97979 |
|  | BC070081 |
|  | BC094761 |
|  | AAH94761 |
|  | BC118665 |
|  | AAI18666 |
|  | BC128419 |
|  | AAI28420 |
|  | CB160831 |
|  | DA371223 |
|  | DC347326 |
|  | DQ088980 |
|  | AAZ66620 |
|  | EF210210 |
|  | ABQ66237 |
|  | EF210211 |
|  | ABQ66238 |
|  | GU228573 |
|  | GU228584 |
|  | GU255993 |
|  | ADL28125 |
|  | HM437234 |
|  | ADN43066 |
|  | HM437235 |
|  | HQ730875 |
|  | HQ912715 |
|  | ADZ75461 |
|  | HQ999997 |
|  | ADY76965 |
|  | K03193 |
|  | AAA52371 |
|  | U48722 |
|  | AAC50802 |
|  | U51732 |
|  | U95089 |
|  | AAB53063 |
|  | X00588 |
|  | CAA25240 |
|  | X00663 |
|  | CAA25282 |
|  | P00533 |
|  | NC\_000077 |
|  | NM\_207655 |
|  | NP\_997538 |
|  | NM\_007912 |
|  | NP\_031938 |
|  | AF125256 |
|  | AAG43241 |
|  | AF456454 |
|  | AAL56615 |
|  | AH009944 |
|  | AAG28045 |
|  | AAG28046 |
|  | AAG28047 |
|  | AL645532 |
|  | BX255277 |
|  | CH466574 |
|  | EDL40666 |
|  | EDL40667 |
|  | HM570131 |
|  | HM570132 |
|  | HM570133 |
|  | HM570134 |
|  | HM570135 |
|  | HM570136 |
|  | HM570137 |
|  | HM570138 |
|  | HM570139 |
|  | HM570140 |
|  | HM570141 |
|  | HM570142 |
|  | HM570143 |
|  | HM570144 |
|  | HM570145 |
|  | HM570146 |
|  | HM570147 |
|  | HM570148 |
|  | HM570149 |
|  | HM570150 |
|  | HM570151 |
|  | HM570152 |
|  | HM570153 |
|  | HM570154 |
|  | HM570155 |
|  | HM570156 |
|  | AF124513 |
|  | AAD44149 |
|  | AF275367 |
|  | AAG24386 |
|  | AF277898 |
|  | AAK01081 |
|  | AK004883 |
|  | BAB23641 |
|  | AK004911 |
|  | BAB23662 |
|  | AK004944 |
|  | BAB23688 |
|  | AK014017 |
|  | AK033431 |
|  | AK049452 |
|  | AK087861 |
|  | AK134262 |
|  | AK163338 |
|  | BAE37306 |
|  | BC023729 |
|  | AAH23729 |
|  | BC030681 |
|  | BQ553815 |
|  | BY725722 |
|  | L06864 |
|  | AAA53029 |
|  | U03425 |
|  | AAA17899 |
|  | X59698 |
|  | CAA42219 |
|  | X78987 |
|  | CAA55587 |
|  | Z12608 |
|  | CAA78249 |
|  | Q01279 |
|  | NC\_005113 |
|  | NM\_031507 |
|  | NP\_113695 |
|  | XM\_008770416 |
|  | XP\_008768638 |
|  | XM\_008770418 |
|  | XP\_008768640 |
|  | XM\_017599073 |
|  | XP\_017454562 |
|  | AC\_000082 |
|  | AABR07016425 |
|  | AABR07016426 |
|  | AABR07016427 |
|  | AABR07072433 |
|  | AAHX01081375 |
|  | AAHX01081376 |
|  | AAHX01081377 |
|  | AAHX01081378 |
|  | AAHX01081379 |
|  | AAHX01081380 |
|  | AAHX01081381 |
|  | AB025197 |
|  | BAA76391 |
|  | AC098180 |
|  | AF142153 |
|  | AAF27540 |
|  | CH473996 |
|  | EDL97895 |
|  | EDL97896 |
|  | AB079120 |
|  | BAF82007 |
|  | FQ209947 |
|  | FQ210224 |
|  | FQ211093 |
|  | HM801041 |
|  | ADT91284 |
|  | HM801042 |
|  | ADT91285 |
|  | M37394 |
|  | AAF14008 |
|  | U52529 |
|  | AAC53049 |
|  | NC\_018918 |
|  | AF125538 |
|  | AF125539 |
|  | AMYH02015730 |
|  | AMYH02015731 |
|  | AC\_000033 |
|  | AAHY01090988 |
|  | AAHY01090989 |
|  | AAHY01090990 |
|  | S50852 |
|  | S50853 |
|  | S50854 |
|  | S50855 |
|  | XM\_008770417 |
|  | XP\_008768639 |
|  | AC\_000139 |
|  | ABBA01011847 |
|  | ABBA01011848 |
|  | ABBA01011849 |
|  | ABBA01011850 |
|  | U48724 |
|  | U48725 |
|  | U48726 |
|  | U48727 |
|  | U48728 |
|  | U48729 |
|  | XM\_006514491 |
|  | XP\_006514554 |
|  | AF275364 |
|  | AF275365 |
|  | AF275366 |
|  | XM\_005271748 |
|  | XP\_005271805 |
|  | XM\_005271746 |
|  | XP\_005271803 |
|  | XM\_005271747 |
|  | XP\_005271804 |
|  | AC\_000068 |
|  | AACC02000089 |
|  | AABR06080997 |
|  | AABR06080998 |
|  | AABR06080999 |
|  | AABR06081000 |
|  | AABR06081001 |

---

|  |  |
| --- | --- |
| Swiss-Prot ID | EGFR\_HUMAN |
|  | EGFR\_MOUSE |

---

|  |  |
| --- | --- |
| Cell Localization | Cell membrane |
|  | Endoplasmic reticulum membrane |
|  | Golgi apparatus membrane |
|  | Nucleus membrane |
|  | Endosome |
|  | Endosome membrane |
|  | Nucleus |
|  | Secreted |
|  | Plasma membrane |

---

|  |  |
| --- | --- |
| Ensembl ID | ENSG00000146648 |
|  | ENSP00000415559.1 |
|  | ENST00000455089.5 |
|  | ENSP00000395243.3 |
|  | ENST00000454757.6 |
|  | ENSP00000275493.2 |
|  | ENST00000275493.7 |
|  | ENSP00000342376.3 |
|  | ENST00000342916.7 |
|  | ENSP00000413843.2 |
|  | ENST00000420316.6 |
|  | ENSP00000345973.2 |
|  | ENST00000344576.6 |
|  | ENSMUSG00000020122 |
|  | ENSMUSP00000099948.3 |
|  | ENSMUST00000102884.9 |
|  | ENSMUSP00000020329.6 |
|  | ENSMUST00000020329.12 |
|  | ENSRNOG00000004332 |
|  | ENSRNOP00000006087.2 |
|  | ENSRNOT00000006087.2 |
|  | ENSP00000275493 |
|  | ENST00000275493 |
|  | ENSP00000342376 |
|  | ENST00000342916 |
|  | ENSP00000413843 |
|  | ENST00000420316 |
|  | ENSP00000345973 |
|  | ENST00000344576 |
|  | ENSMUSP00000099948 |
|  | ENSMUST00000102884 |
|  | ENSMUSP00000020329 |
|  | ENSMUST00000020329 |
|  | ENSRNOP00000006087 |
|  | ENSRNOT00000006087 |
|  | ENSP00000395243 |
|  | ENST00000454757 |
|  | ENSP00000410031 |
|  | ENST00000442591 |

---

|  |  |
| --- | --- |
| MGI ID | MGI:95294 |
|  | 95294 |

---

|  |  |
| --- | --- |
| RGD ID | 2543 |

---

|  |  |
| --- | --- |
| Unigene ID | Hs.488293 |
|  | Hs.605083 |
|  | Mm.8534 |
|  | Mm.420648 |
|  | Mm.439882 |
|  | Rn.37227 |

---

|  |  |
| --- | --- |
| Homologene ID | 74545 |

---

|  |  |
| --- | --- |
| Shape | Stick-vertex |

---
